# Clinical Studies of Bee Venom Acupuncture for Lower Back Pain in the Korean Literature

**DOI:** 10.3390/toxins14080524

**Published:** 2022-07-30

**Authors:** Soo-Hyun Sung, Ji-Eun Han, Hee-Jung Lee, Minjung Park, Ji-Yeon Lee, Soobin Jang, Jang-Kyung Park, Gihyun Lee

**Affiliations:** 1 Department of Policy Development, National Institute of Korean Medicine Development, Seoul 04554, Korea; koyote10010@nikom.or.kr (S.-H.S.); jieun2342@nikom.or.kr (J.-E.H.); leeheejung@nikom.or.kr (H.-J.L.); 2 Center for Development of Innovative Technologies in Korean Medicine, National Institute of Korean Medicine Development, Seoul 04554, Korea; mj.park@nikom.or.kr; 3 Department of Obstetrics & Gynecology, Daejeon Korean Medicine Hospital of Daejeon University, Daejeon 35235, Korea; jyounl@daum.net; 4 Department of Preventive Medicine, College of Korean Medicine, Daegu Haany University, Gyeongsangbuk-do, Gyeongsan 38609, Korea; suebin@nate.com; 5 Department of Korean Medicine Obstetrics and Gynecology, School of Korean Medicine, Pusan National University, Yangsan 50612, Korea; vivat314@pusan.ac.kr; 6 College of Korean Medicine, Dongshin University, Naju 58245, Korea

**Keywords:** bee venom, bee venom acupuncture, lower back pain, clinical studies, complementary and alternative medicine

## Abstract

This study aimed to identify all of the characteristics of bee venom acupuncture (BVA) for the treatment of lower back pain (LBP) that are described in the Korean literature, and to provide English-speaking researchers with bibliometrics. Six Korean electronic databases and sixteen Korean journals on BVA treatment for back pain were searched up to February 2022. This report included and analyzed 64 clinical studies on BVA interventions for back pain and 1297 patients with LBP. The most common disease in patients with back pain was lumbar herniated intervertebral discs (HIVD) of the lumbar spine (L-spine). All studies used bee venom (BV) diluted with distilled water. The concentration of BVA for HIVD of L-spine patients with LBP ranged from 0.01 to 5.0 mg/mL; the dosage per treatment was 0.02–2.0 mL, and for a total session was 0.3–40.0 mL. The most used outcome measure was the visual analogue scale for back pain (n = 45, 70.3%), and most of the papers reported that each outcome measure had a positive effect. Korean clinical studies were typically omitted from the review research, resulting in potential language bias. This study provides clinical cases in Korea for future development and standardization of BVA treatment for back pain.

## 1. Introduction

Lower back pain (LBP) is a highly uncomfortable and often chronic sensation in the back below the lower rib cage and above the gluteal fold [1]. LBP is the most common musculoskeletal condition affecting the adult population, with a worldwide prevalence of 7.5% in 2017 [2,3,4]. It is a major condition leading to disability, affecting work performance and the overall wellbeing of individuals [5,6].

For the treatment of patients with acute LBP, the guidelines recommend reassurance on the favorable prognosis and advice on returning to normal activities, avoiding bedrest, as well as the use of nonsteroidal anti-inflammatory drugs (NSAIDs) and weak opioids for short periods [7]. For the treatment of patients with chronic LBP, the guidelines recommend the use of NSAIDs and antidepressants, exercise therapy, and psychosocial interventions [7]. NSAIDs are the most frequently prescribed medications worldwide, and are widely used in patients with LBP [8]. However, NSAIDs may cause gastrointestinal ulcers, serious cardiovascular events, hypertension, acute renal failure, and worsening of pre-existing heart failure [9]. The most commonly used complementary and alternative medicine (CAM) treatments for LBP are acupuncture, herbal therapies, chiropractic manipulation, massage, yoga, tai chi, and qigong [10].

Bee venom acupuncture (BVA) involves injecting purified and diluted bee venom (BV) into acupuncture points [11]. BVA is commonly used in Asia, Eastern Europe, and South America [12]. BV is mainly used in East Asian countries, including Korea, for pharmacopuncture, which is a traditional medical treatment that combines acupuncture and herbal medicine, unlike traditional acupuncture [13]. According to the National Survey for Traditional Korean Medicine (TKM), pharmacopuncture is used in 22.4% of TKM clinic patients, and BVA is the second most used treatment in pharmacopuncture [14,15].

Two clinical trials in Western databases (e.g., PubMed, Embase, or the Cochrane Central Register of Controlled Trials) investigated the treatment effect of BVA on LBP. In one randomized controlled trial (RCT), the BVA plus NSAIDs group showed a more significant effect on LBP than that of the control group (saline injection plus NSAIDs) [16]. In another study [17], BVA injection showed a more significant effect than that of the normal saline group. A systematic review on BVA for LBP has not yet been published, and one RCT involving LBP was included in a systematic review on musculoskeletal disorders [18]. Korean trials of TKM interventions have usually been published in TKM journals rather than in Western CAM or conventional medicine journals [19]. Thus, identifying Korean clinical studies for inclusion in English-language reviews is difficult [19]. The language barrier increases the risk of language bias [20]. Therefore, we aimed to identify Korean clinical studies on BVA for LBP, and to provide comprehensive information on BV toxins while developing LBP treatment.

## 2. Results

### 2.1. Study Description

**Figure 1:**
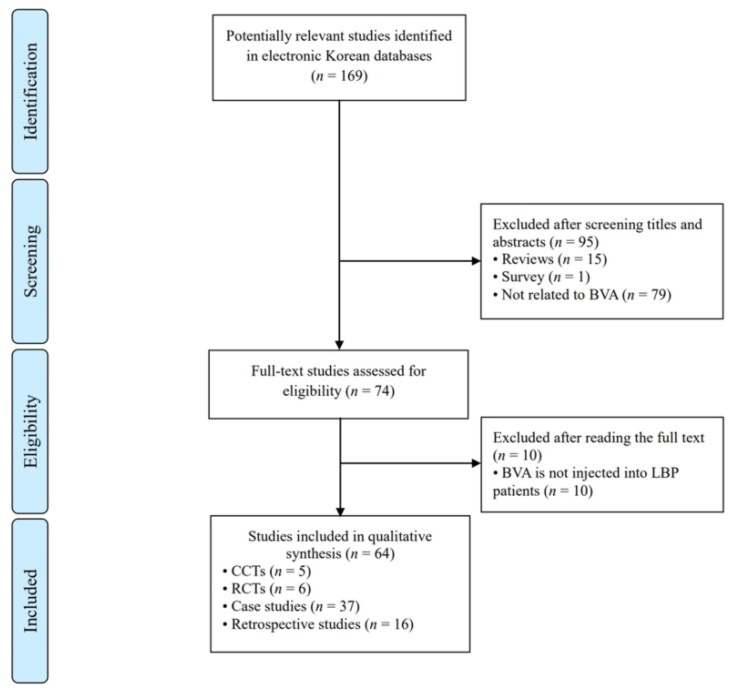
Flowchart of the study selection process. BVA: bee venom acupuncture; CCTs: case-control trials; RCTs: randomized controlled trials.

**Figure 2:**
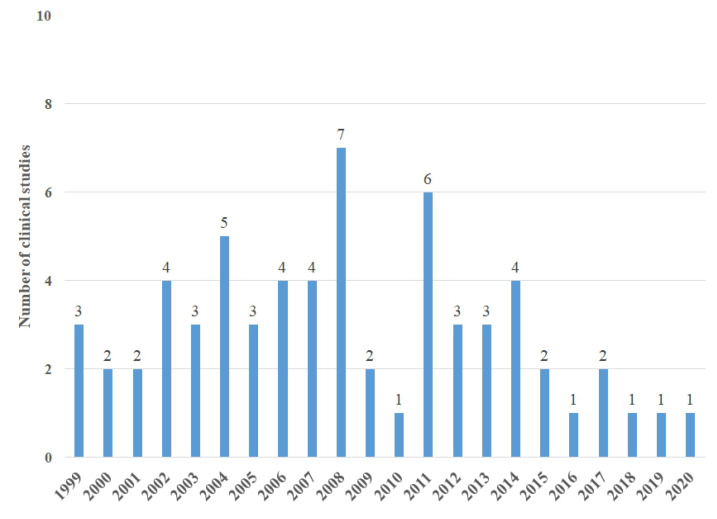
Number of clinical studies in Korea by publication year.

As shown in Figure 1, our search identified 64 full-text articles that met our inclusion criteria [21,22,23,24,25,26,27,28,29,30,31,32,33,34,35,36,37,38,39,40,41,42,43,44,45,46,47,48,49,50,51,52,53,54,55,56,57,58,59,60,61,62,63,64,65,66,67,68,69,70,71,72,73,74,75,76,77,78,79,80,81,82,83,84]. The first BVA-related clinical study published in Korea was published in 1999. From 1999 to 2020, such studies were published yearly, with a maximum of seven papers published in 2008 (Figure 2). The study design is summarized in Table 1. This report includes 37 (57.8%) case studies, 5 (7.8%) case–control trials (CCTs), 6 (9.3%) RCTs, and 16 (25.0%) retrospective studies.

### 2.2. Medical Conditions

Of the 64 included trials, 18 types of single medical conditions were reported in 61 papers, and complex medical conditions were reported in the remaining 3 papers. Six medical conditions—HIVD of L-spine patients with back pain, back pain, failed back surgery syndrome patients with back pain, lumbar spinal stenosis patients with back pain, car accident patients with lower back pain, and back sprain patients with back pain—were mentioned in more than two papers. The numbers of papers and patients by disease are shown in Table 2.

### 2.3. Sample Size

In total, 1295 participants from the 64 clinical studies were included in this review. The sample size per trial ranged from 1 to 208 (20.2 ± 33.1).

### 2.4. BVA Intervention

The intervention used in all included studies was in injection form, using a syringe through which BV was dispensed and injected into the body. The BVA concentration range was 0.01–5.0 mg/mL, and the dosage per treatment and for the total sessions was 0.02–2.0 mL and 0.3–40.0 mL, respectively, for herniated intervertebral disc (HIVD) in lumbar-spine (L-spine) patients with back pain. The BV concentration was 0.05–0.5 mg/mL and the dosage per treatment and the total sessions were 0.03–1.0 mL and 0.51–5.1 mL, respectively, in patients with back pain. The concentration and dosage of BVA according to the participant’s medical condition (e.g., failed back surgery syndrome patients with back pain, lumbar spinal stenosis patients with back pain, car accident patients with LBP, and back sprain patients with back pain) are shown in Table 3. Six papers did not report BV concentration, eight papers did not report the dosage of one session, and eighteen papers did not report the dosage of the total sessions.

### 2.5. Outcome Measures

**Figure 3:**
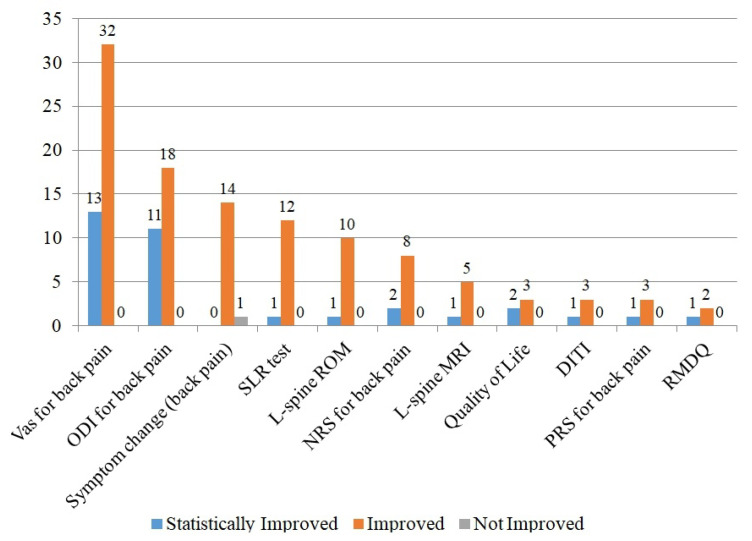
Outcome measures of included clinical studies on BVA for back pain. DITI: digital infrared thermography imaging, MRI: magnetic resonance imaging, ODI: Oswestry disability index, PRS: pain relief scale, RMDQ: Roland–Morris disability questionnaire, ROM: range of motion, SLR: straight leg raise, VAS: visual analogue scale.

A total of 22 types of outcome measures were reported in the 64 included papers. Figure 3 shows the results of classifying the main results of 11 outcome measures used in four or more papers into three categories, including “statistically improved,” “improved”, and “not improved”. The most commonly used outcome measure was the visual analogue scale (VAS) for back pain (n = 45, 70.3%), and most of the papers reported that each evaluation tool had a positive effect.

## 3. Discussion

This study is an analysis of Korean clinical trials published in Korean journals, and we found several clinical studies on BVA for back pain in the Korean literature. The first study on BVA for the treatment of LBP was published in 1999. Since then, such studies have been published yearly until 2020. In Korea, the Ministry of Food and Drug Safety introduced the Good Clinical Practice Guidelines for clinical trials in the late 1990s, and these guidelines seem to have significantly impacted the progress of clinical research, including research on bee venom [19]. In addition, acupuncture for LBP patients in Korea has been reported to reduce the frequency of back surgery, and BVA has been widely used for musculoskeletal disorders (e.g., HIVD, arthritis, back pain, shoulder pain, knee pain, and sprain) [85,86]. Thus, a certain number of BVA clinical trials seem to have been conducted.

Although most studies have reported that BVA is effective for LBP, six studies reported side effects including fever [36], itching [51,52,59,82], local redness [59], edema [59], skin hypersensitivity [73], mild chilling [82], and local rash [82]. BV contains active substances such as peptides, enzymes, and amines, which can exert anti-inflammatory, anti-nociceptive, and anticancer effects, but can also induce neurotoxic symptoms (e.g., redness, swelling, dizziness, nausea, and vomiting) or severe symptoms, such as anaphylaxis [87,88]. Kim et al. [89] suggest that the following are necessary for the safe use of BVA: (1) a qualified or licensed practitioner to treat the patient, and (2) a skin test and post-injection observation in the clinic to manage potential adverse events. Additionally, to develop a treatment using BV for patients with back pain, information on the dosage and concentration is essential to maximize the therapeutic effect while minimizing side effects. Future clinical studies with information on the side effects are necessary.

All 64 Korean clinical trials reported that BV was diluted with saline at a certain ratio and injected into the patients. The BV concentration used for each study was found to cover a wide range, from 0.01 mg/mL to 5.0 mg/mL. In particular, in the case of HIVD in L-spine patients with back pain, the concentration deviation was the largest. When a survey was conducted with 468 TKM doctors, it was reported that the BV concentration used without considering the disease was 0.1–0.3 mg/mL [89]. As such, it can be seen that the deviation of the BV concentration is very large even when compared with the previous study [89]. Based on these basic data, a clinical trial should be established to find the optimal BVA treatment concentration, dose, and frequency for lower back pain.

Pain is mainly evaluated subjectively in patients. Inflammation-related biomarkers, such as interleukin-6, C-reactive protein, and tumor necrosis factor α, along with range of motion (ROM), are also used to measure pain. However, self-reported outcomes, including the VAS, numerical rating scale (NRS), and Oswestry disability index (ODI), are more appropriate to show the clinical effectiveness and patient satisfaction with therapies. Quality of life is also used as an indicator, because LBP lowers the overall physical and psychological health. Although symptom changes in patients confirmed whether the subjectively felt pain of the patient improved, it was not quantified in the same way as when using the VAS. To develop a therapeutic agent, clinical trials that evaluate the effectiveness of the commonly used evaluation tools are necessary.

This study has several limitations. First, this review mostly included case or retrospective studies with low levels of clinical evidence and a relatively small sample size. A higher level of evidence from large-scale clinical studies is needed. Second, the VAS, ODI, and EQ-5D are validated questionnaires, although a meta-analysis was not performed considering the heterogeneity of the included RCTs and the individual variation of the study participants. Third, since this review searched only domestic Korean databases, clinical studies conducted in Korea but published in international journals might have been missing. Finally, the 64 included studies were conducted at university hospitals, and may differ from real-world data obtained at TKM clinics. Therefore, whether this study is representative of the use of BVA for LBP treatment in Korea is difficult to confirm. Nonetheless, many cases of BVA application for the treatment of back pain in Korea exist; the details of BVA summarized in this review could provide information to help in planning clinical trials for new drug development.

## 4. Conclusions

This study showed the clinical research trend for BVA’s use in LBP treatment as published in Korean journals. BV was diluted to an appropriate concentration for clinical purposes, and was confirmed to be an effective treatment for patients with LBP. However, no side effects were reported in most studies, and large variations in the concentration, dose, and number of BVA treatments were noted. This study provides clinical evidence for the future drug development and standardization of LBP treatment using BVA.

## 5. Materials and Methods

### 5.1. Data Sources and Searches

We searched six Korean bibliographic databases (the Korea Institute of Science and Technology Information, the Korean Traditional Knowledge Portal, KoreaMed, OASIS, RISS, and the National Library of Korea) up to February 2022. The Korean trials indexed in non-Korean databases such as PubMed and Embase were not considered.

The search terms were as follows: “bee venom OR bee toxin OR apitherapy OR bee venom therapy OR bee venom acupuncture” AND “back pain” AND “clinical studies OR clinical trial”.

### 5.2. Study Selection

We included all clinical studies (e.g., case studies, case series, CCTs, and RCTs) that evaluated the effects of BVA on back pain. All patients with back pain and without age- or sex-based restrictions were included. We included all types of BVA and all outcome measures (e.g., pain score, symptom change, quality of life, ROM, and adverse events) used for treating back pain. Non-clinical trials—including animal studies, experimental studies, surveys, and reviews—were excluded.

### 5.3. Data Extraction

Three authors (J.-E.H, H.-J.L., and J.-Y.L.) independently extracted data using a predefined data extraction form. Two independent reviewers (S.-H.S. and M.P.) collected data regarding author information, study design, sample size, medical conditions, interventions (i.e., form, concentration, treatment sessions, and dosage), adverse events, outcome measures, and main results. In cases of insufficient outcome data, the corresponding authors were contacted whenever possible. Any disagreements were resolved through discussions with G.L.

## Figures and Tables

**Table 1:** Characteristics of clinical studies of bee venom acupuncture for lower back pain in the Korean literature.

First Author	Study Design	Number of Patients	Medical Conditions	Intervention (Form, Concentration, Treatment Sessions and Dosage)	Adverse Events	Outcome Measure	Main Result
Lee (1999) [21]	Retrospective study	*n* = 12	HIVD of L-spine patients with back pain	1. Form: injection 2. Concentration: 0.5 mg/mL 3. 1 session: n.r. 4. Total 1–9 sessions: n.r.	n.r.	1. Symptom change (back pain)	1. Improved
Kim (1999) [22]	Retrospective study	*n* = 22	HIVD of L-spine patients with back pain	1. Form: injection 2. Concentration: 0.05 mg/mL 3. 1 session: 0.9 mL 4. Total 8 sessions: 7.2 mL	n.r.	1. Symptom change (back pain) 2. L-spine MRI (degree of HIVD) 3. Satisfaction of patients	1. Improved 2. Positive ^a^ 3. Improved
Park (1999) [23]	Case studies	*n* = 100	Patients with lower back pain	1. Form: injection 2. Concentration: n.r. 3. 1 session: n.r. 4. Total session and dose: n.r.	n.r.	1. SLR test	1. Improved
Lee (2000) [24]	Case studies	*n* = 18	Patients with back pain (degenerative arthritis and HIVD of L-spine)	1. Form: injection 2. Concentration: n.r. 3. 1 session: n.r. 4. Total 21–64 sessions: n.r.	None	1. Symptom change (back pain)	1. Improved
Yun (2000) [25]	Case studies	*n* = 1	HIVD of L-spine patients with back pain	1. Form: injection 2. Concentration: 0.3 mg/mL 3. 1 session: 1 mL 4. Total 20 sessions: 20 mL	n.r.	1. VAS for back pain 2. L-spine ROM	1. Improved 2. Improved
Kim (2001) [26]	Case studies	*n* = 19	Patients with back pain (myofascial pain syndrome, HIVD of L-spine, degenerative spondylitis, and ankylosing spondylitis)	1. Form: injection 2. Concentration: n.r. 3. 1 session: n.r. 4. Total 1–20 sessions: n.r.	n.r.	1. Symptom change (back pain)	1. Improved in 12 cases, not improved in 7 cases
Lee (2001) [27]	Case studies	*n* = 1	HIVD of L-spine patient with back pain	1. Form: injection 2. Concentration: 0.03 mg/mL 3. 1 session: 0.5–1.4 mL 4. Total 13 sessions: 13.7 mL	n.r.	1. VAS for back pain 2. L-spine ROM 3. L-spine CT (degree of HIVD)	1. Improved 2. Improved 3. Not improved
Lim (2002) [28]	Case studies	*n* = 1	Sequestrated disc patient with back pain	1. Form: injection 2. Concentration: 0.25 mg/mL 3. 1 session: 0.4–2.0 mL 4. Total 20 sessions: 8–40 mL	n.r.	1. VAS for back pain 2. ODI for back pain 3. L-spine ROM	1. Improved 2. Improved 3. Improved
Yoo (2002) [29]	Case studies	*n* = 1	Klippel–Trenaunay–Weber syndrome patient with back pain	1. Form: injection 2. Concentration: 0.5 mg/mL 3. 1 session: 0.2–0.4 mL 4. Total 10 sessions: 2.0–4.0 mL	n.r.	1. Symptom change (back pain) 2. DITI of back	1. Improved 2. Improved
Bae (2002) [30]	Case studies	*n* = 1	HIVD of L-spine patients with back pain	1. Form: injection 2. Concentration: 0.5 mg/mL 3. 1 session: n.r. 4. Total session and dosage: n.r.	n.r.	1. SLR Test 2. Symptom change (back pain)	1. Improved 2. Improved
Moon (2002) [31]	Case studies	*n* = 1	Diffuse idiopathic skeletal hyperostosis patient with back pain	1. Form: injection 2. Concentration: 0.1 mg/mL and 0.5 mg/mL 3. 1 session: 0.3–1.2 mL 4. Total session and dose: n.r.	n.r.	1. SLR Test 2. Symptom change (back pain)	1. Improved 2. Improved
Jun (2003) [32]	RCT	*n* = 45	HIVD of L-spine patients with back pain	1. Form: injection 2. Concentration: 0.16 mg/mL 3. 1 session: 0.1–1.0 mL 4. Total above 12 sessions: above 1.2–12 mL	n.r.	1. VAS for back pain 2. PRS for back pain 3. DITI of back	1. Positive ^b^ 2. Positive ^b^ 3. Positive ^b^
Chung (2003) [33]	Retrospective study	*n* = 24	HIVD of L-spine patients with back pain	1. Form: injection 2. Concentration: 0.05 mg/mL (1–2 visit), 0.1 mg/mL (3 visit), 0.2 mg/mL (4 visit), 0.4 mg/mL (5–6 visit) 3. 1 session: 0.05 mL 4. Total 6 sessions: 0.3 mL	n.r.	1. VAS for back pain 2. ODI for back pain 3. L-spine ROM	1. Positive ^b^ 2. Positive ^b^ 3. Positive ^a^
Hwang (2003) [34]	Case studies	*n* = 1	Spinal meningeal cyst patient with back pain	1. Form: injection 2. Concentration: 0.16 mg/mL 3. 1 session: 0.08 mL 4. Total 21 sessions: 1.68 mL	n.r.	1. VAS for back pain 2. PRS for back pain 3. L-spine MRI (degree of HIVD) 4. L-spine ROM	1. Improved 2. Improved 3. Improved 4. Improved
Cha (2004) [35]	CCT	*n* = 29	HIVD of L-spine patients with back pain	1. Form: injection 2. Concentration: 0.1 mg/mL, 0.25 mg/mL or 0.5 mg/mL 3. 1 session: 0.05 mL 4. Total 7 sessions: 0.35 mL	n.r.	1. VAS for back pain 2. ODI for back pain	1. Positive ^c^ 2. Positive ^c^
Lee (2004) [36]	Retrospective study	*n* = 20	HIVD of L-spine patients with back pain	1. Form: injection 2. Concentration: 0.05 mg/mL or 0.25 mg/mL 3. 1 session: 0.1–1.5 mL 4. Total 9 sessions: 0.9–13.5 mL	Fever in 3 cases	1. VAS for back pain 2. Grade classification of recovery degree	1. Positive ^c^ 2. Improved
Lee (2004) [37]	Case studies	*n* = 1	Failed back surgery syndrome patient with back pain	1. Form: injection 2. Concentration: 0.125 mg/mL 3. 1 session: 0.3–1 mL 4. Total 10 sessions: 8.4 mL	n.r.	1. VAS for back pain 2. ODI for back pain 3. Physical examination	1. Improved 2. Improved 3. Improved
Lee (2004) [38]	Case studies	*n* = 1	Causalgia patient after lumbar partial laminectomy with back pain	1. Form: injection 2. Concentration: 0.5 mg/mL 3. 1 session: 0.3 mL 4. Total 44 sessions: 13.2 mL	n.r.	1. VAS for back pain	1. Improved
Yoo (2004) [39]	Case studies	*n* = 1	HIVD of L-spine patient with back pain	1. Form: injection 2. Concentration: 0.05 mg/mL 3. 1 session: 0.3–0.6 mL 4. Total 22 sessions: n.r.	n.r.	1. VAS for back pain	1. Improved
Kim (2005) [40]	Case studies	*n* = 1	Neurogenic bladder after lumbar disc herniation with back pain	1. Form: injection 2. Concentration: 0.5 mg/mL 3. 1 session: 0.2–1.0 mL 4. Total 17 sessions: 12.4 mL	n.r.	1. VAS for back pain 2. Physical examination	1. Improved 2. Improved
Kim (2005) [41]	Retrospective study	*n* = 15	HIVD of L-spine patients with back pain	1. Form: injection 2. Concentration: 0.5 mg/mL, 0.25 mg/mL or 0.1 mg/mL 3. 1 session: 0.1–1 mL 4. Total session and dosage: n.r.	n.r.	1. VAS for back pain 2. Symptom change (back pain)	1. Improved 2. Improved
Kim (2005) [42]	RCT	*n* = 30	Back sprain patients with back pain	1. Form: injection 2. Concentration: 0,3 mg/mL 3. 1 session: 1 mL 4. Total 5 sessions: 5 mL	n.r.	1. VAS for back pain 2. ODI for back pain	1. Improved 2. Improved
Lee (2006) [43]	Case studies	*n* = 1	HIVD of L-spine patients with back pain	1. Form: injection 2. Concentration: 0.3 mg/mL 3. 1 session: 0.8 mL 4. Total 6 sessions: 4.8 mL	n.r.	1. VAS for back pain 2. L-spine ROM	1. Improved 2. Improved
Cha (2006) [44]	CCT	*n* = 18	HIVD of L-spine patients with back pain	1. Form: injection 2. Concentration: 0.25 mg/mL 3. 1 session: 0.2–1.4 mL 4. Total 2–15 sessions: 0.4–21 mL	n.r.	1. VAS for back pain 2. L-spine ROM	1. Improved 2. Improved
Yu (2006) [45]	Retrospective study	*n* = 35	HIVD of L-spine patients with back pain	1. Form: injection 2. Concentration: 0.25 mg/mL or 0.5 mg/mL 3. 1 session: 0.1–1.0 mL 4. Total session and dosage: n.r.	n.r.	1. VAS for back pain 2. ODI for back pain 3. SLR test 4. L-spine ROM 5. Symptom change (back pain)	1. Improved 2. Improved 3. Improved 4. Improved 5. Improved
Kang (2006) [46]	Case studies	*n* = 1	Patients with lower back pain (lumbar spinal stenosis and HIVD of L-spine)	1. Form: injection 2. Concentration: 0.3 mg/mL or 2 mg/mL 3. 1 session: 0.03–0.3 mL 4. Total 17 sessions: 0.51–5.1 mL	n.r.	1. VAS for back pain	1. Improved
Lee (2007) [47]	Case studies	*n* = 1	Lumbar spinal stenosis patients with back pain	1. Form: injection 2. Concentration: 0.3 mg/mL 3. 1 session: 0.04 mL 4. Total 20 sessions: 0.8 mL	n.r.	1. ODI for back pain 2. VAS for back pain 3. L-spine MRI (degree of stenosis)	1. Improved 2. Improved 3. Improved
Lee (2007) [48]	Retrospective study	*n* = 10	HIVD of L-spine patients with back pain	1. Form: injection 2. Concentration: 0.1 mg/mL 3. 1 session: 0.6 mL 4. Total 4 sessions: 2.4 mL	n.r.	1. VAS for back pain 2. SLR test	1. Improved 2. Improved
Seo (2007) [49]	Case studies	*n* = 3	HIVD of L-spine patients with back pain	1. Form: injection 2. Concentration: 0.005–5.0 mg/mL 3. 1 session: 0.08–0.2 mL 4. Total 2–10 sessions: 0.32–0.1 mL	n.r.	1. VAS for back pain 2. PRS for back pain 3. SLR test	1. Improved 2. Improved 3. Improved
Lee (2007) [50]	Retrospective study	*n* = 60	HIVD of L-spine patients with back pain	1. Form: injection 2. Concentration: 0.05 mg/mL 3. 1 session: n.r. 4. Total session and dosage: n.r.	n.r.	1. VAS for back pain 2. Symptom change (back pain) 3. SLR test	1. Improved 2. Improved 3. Improved
Kim (2008) [51]	RCT	*n* = 19	Patients with lower back pain	1. Form: injection 2. Concentration: 0.1 mg/mL 3. 1 session: 0.5 mL (1 and 2 visit), 0.7 mL(3 and 4 visit) 4. Total 4 sessions: 2.4 mL	Itching in 0.85 ± 1.72 cases	1. VAS for back pain 2. ODI for back pain	1. Positive ^c^ 2. Positive ^c^
Kwon (2008) [52]	Retrospective study	*n* = 13	HIVD of L-spine patients with back pain	1. Form: injection 2. Concentration: 0.5 mg/mL 3. 1 session: 0.2 mL 4. Total 12 sessions: 2.4 mL	Itching in 8 cases	1. VAS for back pain 2. RMDQ	1. Positive ^a^ 2. Positive ^a^
Youn (2008) [53]	Retrospective study	*n* = 20	HIVD of L-spine patients with back pain	1. Form: injection 2. Concentration: 0.125 mg/mL 3. 1 session: 1 mL 4. Total 7 sessions: 7 mL	n.r.	1. VAS for back pain 2. ODI for back pain 3. Quality of life (SF-36)	1. Positive ^a^ 2. Positive ^b^ 3. Positive ^b^
Youn (2008) [54]	Retrospective study	*n* = 36	HIVD of L-spine patients with back pain	1. Form: injection 2. Concentration: 0.125 mg/mL 3. 1 session: 1 mL 4. Total 8 sessions: 8 mL	n.r.	1. VAS for back pain 2. ODI for back pain 3. Quality of life (SF-36)	1. Positive ^a^ 2. Positive ^a^ 3. Positive ^a^
Cho (2008) [55]	Case studies	*n* = 1	Baastrup’s disease patient with back pain	1. Form: injection 2. Concentration: 0.125 mg/mL 3. 1 session: 1 mL 4. Total 34 sessions: 34 mL	n.r.	1. VAS for back pain 2. ODI for back pain	1. Improved 2. Improved
Jeong (2008) [56]	Case studies	*n* = 16	Lumbar spinal stenosis patients with back pain	1. Form: injection 2. Concentration: 0.2 mg/mL or 0.5 mg/mL 3. 1 session: 0.8–1.0 mL 4. Total session and dosage: n.r.	n.r.	1. VAS for back pain 2. ODI for back pain 3. Symptom change (back pain)	1. Improved 2. Improved 3. Improved
Kim (2008) [57]	CCT	*n* = 33	Lumbar hyperlordosis patient with back pain	1. Form: injection 2. Concentration: 0.16 mg/mL 3. 1 session: n.r. 4. Total session and dosage: n.r.	n.r.	1. VAS for back pain 2. ODI for back pain 3. L-spine X-ray (degree of hyperlordosis)	1. Improved 2. Improved 3. Improved
Kwon (2009) [58]	Retrospective study	*n* = 35	HIVD of L-spine patients with back pain	1. Form: injection 2. Concentration: 0.125 mg/mL 3. 1 session: 1 mL 4. Total 24 sessions: 24 mL	n.r.	1. VAS for back pain 2. ODI for back pain 3. L-spine CT (degree of HIVD)	1. Positive ^a^ 2. Positive ^a^ 3. Improved
Yu (2009) [59]	Case studies	*n* = 1	Failed back surgery syndrome patient with back pain	1. Form: injection 2. Concentration: 0.3 mg/mL or 0.5 mg/mL 3. 1 session: 0.02–0.5 mL 4. Total 9 sessions: 2.5 mL	Local redness, itching, and edema in 1 case	1. VAS for back pain 2. Symptom change (back pain)	1. Improved 2. Improved
Lee (2010) [60]	Case studies	*n* = 3	Failed back surgery syndrome patient with back pain	1. Form: injection 2. Concentration: 0.125 mg/mL or 0.25 mg/mL 3. 1 session: 0.2–1 mL 4. Total 18–34 sessions: 3.6–34 mL	n.r.	1. NRS for back pain 2. Physical examination	1. Improved 2. Improved
Lee (2011) [61]	RCT	*n* = 34	Car accident patients with lower back pain	1. Form: injection 2. Concentration: 0.05 mg/mL or 0.1 mL/mL 3. 1 session: 0.2–1.0 mL 4. Total 8 sessions: 1.6–8.0 mL	n.r.	1. VAS for back pain 2. ODI for back pain	1. Positive ^b^ 2. Positive ^a^
Lim (2011) [62]	Case studies	*n* = 1	Failed back surgery syndrome patient with back pain	1. Form: injection 2. Concentration: 0.01 mg/mL or 0.25 mg/mL 3. 1 session: 0.6 mL 4. Total 11–13 sessions: 6.6–7.8 mL	n.r.	1. VAS for back pain 2. ODI for back pain 3. SF-MPQ	1. Improved 2. Improved 3. Improved
Shin (2011) [63]	CCT	*n* = 36	Back sprain patients with back pain	1. Form: injection 2. Concentration: 0.1 mg/mL 3. 1 session: 0.1 mL 4. Total 8–13 sessions: 0.8–25 mL	n.r.	1. VAS for back pain	1. Positive ^c^
Han (2011) [64]	Case studies	*n* = 119	Lumbar spinal stenosis patients with back pain	1. Form: injection 2. Concentration: 0.13 mg/mL or 0.25 mg/mL 3. 1 session: 0.8–1.0 mL 4. Total session and dosage: n.r.	n.r.	1. NRS for back pain 2. ODI for back pain 3. Symptom change (back pain)	1. Improved 2. Improved 3. Improved
Shin(2011) [65]	RCT	*n* = 34	HIVD of L-spine patients with back pain	1. Form: injection 2. Concentration: 0.1 mg/mL or 0.25 mg/mL 3. 1 session: 0.2–1.0 mL 4. Total session and dosage: n.r.	n.r.	1. VAS for back pain 2. Aberdeen LBP scale	1. Improved 2. Improved
Cho (2011) [66]	Case studies	*n* = 30	Failed back surgery syndrome patient with back pain	1. Form: injection 2. Concentration: 0.05 mg/mL, 0.1 mg/mL or 0.5 mg/mL 3. 1 session: 0.4–1.0 mL 4. Total session and dosage: n.r.	n.r.	1. NRS for back pain 2. Symptom change 3. SLR test	1. Improved 2. Improved 3. Improved
Ro (2012) [67]	RCT	*n* = 30	Spondylolisthesis patients with back pain	1. Form: injection 2. Concentration: 0.1 mg/mL 3. 1 session: 0.2–1.0 mL 4. Total 14 sessions: 2.8–14 mL	n.r.	1. NRS for back pain 2. ODI for back pain	1. Positive ^c^ 2. Positive ^c^
Kim (2012) [68]	CCT	*n* = 20	Car accident patients with lower back pain	1. Form: injection 2. Concentration: 0.1 mg/mL 3. 1 session: 1.0 mL 4. Total 8 sessions: 8.0 mL	n.r.	1. VAS for back pain 2. Patient condition grade 3. Five-point Likert scale	1. Improved 2. Improved 3. Improved
Yeon (2012) [69]	Case studies	*n* = 2	Patients with lower back pain	1. Form: injection 2. Concentration: 0.05 mg/mL 3. 1 session: 0.3 mL 4. Total 1 session: 0.3 mL	n.r.	1. VAS for back pain 2. L-spine ROM 3. SLR test	1. Improved 2. Improved 3. Improved
Jung (2013) [70]	Retrospective study	*n* = 208	HIVD of L-spine patients with back pain	1. Form: injection 2. Concentration: 0.1 mg/mL 3. 1 session: 1 mL 4. Total 8–32 sessions: 8–32 mL	n.r.	1. NRS for back pain 2. ODI for back pain 3. SLR test 4. L-spine ROM	1. Positive ^c^ 2. Positive ^c^ 3. Positive ^c^ 4. Positive ^c^
Ji (2013) [71]	Case studies	*n* = 1	Lumbar spinal stenosis patients with back pain	1. Form: injection 2. Concentration: 0.1 mg/mL 3. 1 session: 0.8 mL 4. Total 18 sessions: 14.4 mL	n.r.	1. VAS for back pain 2. Start time of claudication 3. DITI of back	1. Improved 2. Improved 3. Improved
Park (2013) [72]	Retrospective study	*n* = 10	HIVD of L-spine patients with back pain	1. Form: injection 2. Concentration: 0.1 mg/mL 3. 1 session: 1 mL 4. Total session and dosage: n.r.	n.r.	1. VAS for back pain 2. PRS for back pain 3. ODI for back pain 4. DITI of back	1. Improved 2. Improved 3. Improved 4. Improved
Lee (2014) [73]	Retrospective study	*n* = 62	Patients with lower back pain	1. Form: injection 2. Concentration: 0.05 mg/mL 3. 1 session: 0.1–0.6 mL 4. Total 6 sessions: 0.6–2.1 mL	Skin hypersensitivity (edema, rash, and itching) in 22 cases	1. VAS for back pain 2. ODI for back pain	1. Positive ^a^ 2. Positive ^c^
Kim(2014) [74]	Case studies	*n* = 1	Cauda equina syndrome patient with back pain	1. Form: injection 2. Concentration: 0.1 mg/mL 3. 1 session: 0.5–2.0 mL 4. Total 18 sessions: 9–36 mL	n.r.	1. Symptom change (back pain) 2. L-spine MRI (cauda equine syndrome)	1. Improved 2. Improved
Kim (2014) [75]	Case studies	*n* = 1	HIVD of L-spine and femoroacetabular impingement patient with back pain	1. Form: injection 2. Concentration: 0.1 mg/mL 3. 1 session: 0.1–0.3 mL 4. Total 35 sessions: 3.5–10.5 mL	n.r.	1. NRS for back pain 2. ODI for back pain 3. SLR test 4. Quality of life (EQ-5D)	1. Improved 2. Improved 3. Improved 4. Improved
Kwon (2014) [76]	Case studies	*n* = 1	HIVD of L-spine patients with back pain	1. Form: injection 2. Concentration: 0.1 mg/mL 3. 1 session: 0.7 mL 4. Total 7 sessions: 4.9 mL	n.r.	1. VAS for back pain 2. ODI for back pain 3. L-spine MRI (degree of HIVD)	1. Improved 2. Improved 3. Improved
Ji (2015) [77]	Case studies	*n* = 1	Back pain patient after decompression of traumatic compartment syndrome	1. Form: injection 2. Concentration: 0.05 mg/mL 3. 1 session: 0.4 mL 4. Total 63 sessions: 25.2 mL	n.r.	1. VAS for back pain 2. L-spine ROM	1. Improved 2. Improved 3. Improved
Yang (2015) [78]	Case studies	*n* = 1	HIVD of L-spine patients with back pain	1. Form: injection 2. Concentration: 0.1 mg/mL 3. 1 session: n.r. 4. Total session and dosage: n.r.	None	1. VAS for back pain 2. ODI for back pain	1. Improved 2. Improved
Kim (2016) [79]	Retrospective study	*n* = 40	Patients with lower back pain	1. Form: injection 2. Concentration: n.r. 3. 1 session: 0.5 mL 4. Total 8 sessions: 4.0 mL	n.r.	1. VAS for back pain 2. ODI for back pain	1. Positive ^a^ 2. Positive ^a^
Ok (2017) [80]	Case studies	*n* = 2	HIVD of L-spine patients with back pain	1. Form: injection 2. Concentration: n.r. 3. 1 session: 1.5 mL 4. Total 12–16 sessions: 18–24 mL	n.r.	1. NRS for back pain 2. SLR test 3. RMDQ	1. Improved 2. Improved 3. Improved
Nam (2017) [81]	Case studies	*n* = 4	HIVD of L-spine patients with back pain	1. Form: injection 2. Concentration: 0.05 mg/mL 3. 1 session: 1 mL 4. Total 2–8 sessions: 2–8 mL	n.r.	1. VAS for back pain 2. ODI for back pain	1. Improved 2. Improved
Hwang (2018) [82]	Case studies	*n* = 2	HIVD of L-spine patients with back pain	1. Form: injection 2. Concentration: 0.1 mg/mL 3. 1 session: 0.1–0.3 mL 4. Total 5–8 sessions: 0.5–2.4 mL	Mild chilling, local rash, itching in 2 cases	1. NRS for back pain 2. ODI for back pain 3. RMDQ	1. Improved 2. Improved 3. Improved
Ryu (2019) [83]	Case studies	*n* = 1	HIVD of L-spine patients with back pain	1. Form: injection 2. Concentration: n.r. 3. 1 session: 0.2–1.5 mL 4. Total 16 sessions: 20.5 mL	n.r.	1. NRS for back pain 2. ODI for back pain 3. L-spine MRI (degree of HIVD) 4. SLR test 5. Quality of life (EQ-5D)	1. Improved 2. Improved 3. Improved 4. Improved 5. Improved
Bong (2020) [84]	Case studies	*n* = 3	Patients with lower back pain	1. Form: injection 2. Concentration: 0.1 mg/mL 3. 1 session: 1 mL 4. Total 8–9 sessions: 8–9 mL	None	1. NRS for back pain 2. ODI for back pain 3. Quality of life (EQ-5D)	1. Improved 2. Improved 3. Improved

^a^*p* < 0.05; ^b^ *p* < 0.01; ^c^ p < 0.001. CT: computed tomography, DITI: digital infrared thermography imaging, EQ-5D: EuroQol 5-Dimensional, L-spine: lumbar spine, MRI: magnetic resonance imaging, NRS: numeral rating scale, ODI: Oswestry disability index, n.r.: not reported, PRS: pain relief scale, RMDQ: Roland–Morris disability questionnaire, ROM: range of motion, LBP: lower back pain, SF-36: 36-item short-form survey, SF-MPQ: short-form McGill pain questionnaire, SLR: straight leg raise, VAS: visual analogue scale.

**Table 2:** Numbers of papers and patients according to medical condition.

Medical Conditions	Number of Papers (N (%))	Number of Patients (Mean)
HIVD of L-spine patients with back pain	30 (47.7)	22.17 ± 38.4
Back pain	6 (9.2)	37.7 ± 38.2
Failed back surgery syndrome patients with back pain	5 (7.7)	7.2 ± 12.8
Lumbar spinal stenosis patients with back pain	4 (6.2)	34.3 ± 56.9
Car accident patients with lower back pain	2 (3.1)	27 ± 9.9
Back sprain patients with back pain	2 (3.1)	33 ± 4.2

HIVD: herniated intervertebral disc.

**Table 3:** Concentration and dosage of BV according to participants’ medical conditions.

Conditions of Participants	Concentration (mg/mL)	Dosage	
		Dosage Per 1 Session (mL)	Dosage for Total Session (mL)
HIVD of L-spine patients with back pain	0.01–5.0	0.02–2.0	0.3–40.0
Back pain	0.05–0.5	0.03–1.0	0.51–5.1
Failed back surgery syndrome patients with back pain	0.05–0.25	0.1–2.0	0.5–21
Lumbar spinal stenosis patients with back pain	0.05–0.5	0.3–1.2	14.4
Car accident patients with lower back pain	0.1–0.3	0.5–0.8	0.35–4.8
Back sprain patients with back pain	0.05	0.9	7.2

HIVD: herniated intervertebral disc.

## Data Availability

The datasets (Korean clinical studies) used and/or analyzed during this study are available from the corresponding author upon reasonable request.
